# Repeated Stimulus Exposure Alters the Way Sound Is Encoded in the Human Brain

**DOI:** 10.1371/journal.pone.0010283

**Published:** 2010-04-22

**Authors:** Kelly L. Tremblay, Kayo Inoue, Katrina McClannahan, Bernhard Ross

**Affiliations:** 1 Department of Speech and Hearing Sciences, University of Washington, Seattle, Washington, United States of America; 2 Rotman Research Institute, Toronto, Ontario, Canada; University of Leuven, Belgium

## Abstract

Auditory training programs are being developed to remediate various types of communication disorders. Biological changes have been shown to coincide with improved perception following auditory training so there is interest in determining if these changes represent biologic markers of auditory learning. Here we examine the role of stimulus exposure and listening tasks, in the absence of training, on the modulation of evoked brain activity. Twenty adults were divided into two groups and exposed to two similar sounding speech syllables during four electrophysiological recording sessions (24 hours, one week, and up to one year later). In between each session, members of one group were asked to identify each stimulus. Both groups showed enhanced neural activity from session-to-session, in the same P2 latency range previously identified as being responsive to auditory training. The enhancement effect was most pronounced over temporal-occipital scalp regions and largest for the group who participated in the identification task. The effects were rapid and long-lasting with enhanced synchronous activity persisting months after the last auditory experience. Physiological changes did not coincide with perceptual changes so results are interpreted to mean stimulus exposure, with and without being paired with an identification task, alters the way sound is processed in the brain. The cumulative effect likely involves auditory memory; however, in the absence of training, the observed physiological changes are insufficient to result in changes in learned behavior.

## Introduction

Understanding the effects of sensory experience on the brain is a long-standing theme of research, crossing all modalities, in the field of neuroscience. In the auditory domain, motivation comes from at least two streams of scientific inquiry: 1) defining normal processes associated with auditory learning; including, but not limited to speech, language, and music; and 2) using the proposed models of learning to develop effective ways of (re)habilitating impaired perception.

One current area of interest is to better define the perceptual and physiological effects of sound exposure. From bird song to infant language development and from second language learning to relearning to hear after a period of deafness, it is important to know if being exposed to sound is sufficient to alter the physiological representation and perception of sound [1], [2]. While there is much evidence documenting the perceptual gains and coincident physiological changes that take place when sound is paired with a training task, to form some type of meaning or purpose to the listening experience, less is known about the effects of mere stimulus exposure on the central auditory system (for reviews see [1]–[4]).

Here we examine the effects of repeated stimulus exposure as well as listening tasks, in the absence of training, on the human central auditory system. Electroencephalography (EEG) tools are used because they are non-invasive and sensitive to experience-related changes in the central auditory system. In particular, electro- and magneto- encephalography recordings of the P1-N1-P2 complex have been used to examine the effects of tone [5], [6], speech [7]–[12], and musical training [13]–[19] on patterns of auditory evoked potentials (AEPs) and/or neuromagnetic fields (AEFs). One common finding is that increased P2 amplitude coincides with increased experience with, and/or improved perception of, the trained stimuli. A typical interpretation of these results is that auditory training alters the physiological representation of the cue being trained and that these physiological changes reflect learning-related plastic changes in the human central auditory system (for a review, see [2]). More specifically, changes in scalp recorded evoked potentials are presumed to reflect changes in the amplitude and/or synchrony of local field potentials caused by transmembrane currents in large numbers of neurons.

Inherent in any type of training paradigm, however, is stimulus exposure, focused attention, and decision making [20]. It is therefore possible that changes in P2 amplitude reflect any one or combination of these processes, independent of coinciding perceptual gains. As an example, Sheehan et al. [10] reported increased P2 amplitudes in two groups of subjects; a group that participated in training exercises as well as the control group that did not. Because enhanced P2 amplitudes were seen in the untrained group, Sheehan et al. concluded that mere stimulus exposure, rather than training, was responsible for the increases in P2 amplitude.

In the Sheehan et al. [10] example, the untrained participants were tested and then retested one week later. This type of repeated measures design is similar to that used in test-retest reliability studies and numerous studies have shown good test-retest reliability for N1 and P2 responses regardless of whether test-retest sessions took place within a week, or within the year [7], [21]–[24]. Results from these reliability studies imply that stimulus experience during one test session does not automatically affect the physiological representation of sound during a second test session, despite conclusions made by Sheehan et al. However, the majority of test-retest studies reported amplitude measurements from a single midline electrode site (e.g., Cz), or a small subset of midline electrodes, and possibly missed effects that were not identifiable when looking at a limited region of the scalp. This point is bolstered by recent data reported by Ross and Tremblay [25].

Ross and Tremblay [25], reported increases in P2 amplitude, from one test session to another, in the absence of training, when examining source waveforms that were generated using a 151-channel whole-head neuromagnetometer (MEG). There was little change in P2 with repeated stimulus presentations within a single test session; however, enhanced P2 activity was seen between test sessions with the second session taking place on a separate day. In the experiments by Ross and Tremblay [25] and Sheehan et al. [10] participants took part in a perceptual task following the initial and final MEG/EEG recording sessions so that pre- and post-training perceptual performance could be compared. Even though feedback was not provided during these perceptual tests, it is possible that the task provides meaning/purpose to the otherwise irrelevant sound stimuli and activates brain processes that are later manifested in enhanced P2 amplitudes. Hence, the questions posed in the present study are: Does a perceptual task performed during Session 1, affect the physiological processing of sound during Session 2? Does repeated stimulus exposure, in the absence of training, enhance P2 amplitudes?

**Figure 1:**
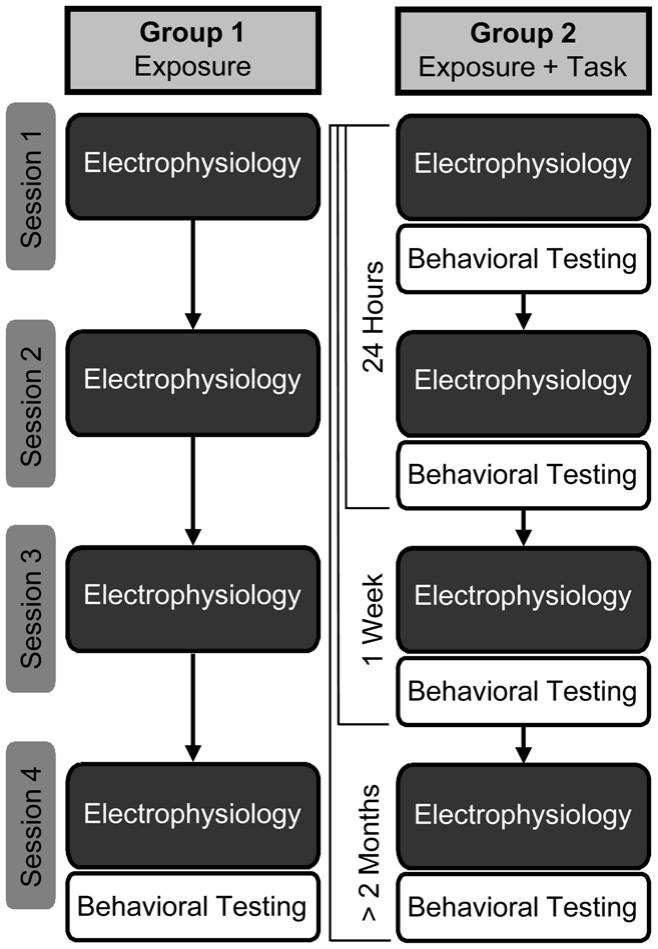
Flowchart describing the procedure.

Using well established stimuli and tasks from voice-onset time categorical perception learning experiments ([26]; see [2] for review) participants heard two variants of the speech syllable “ba”. Without training, native English speakers cannot identify the 10 ms pre-voiced cue that distinguishes the two sounds and both are typically described as sounding like “ba”. With training and feedback, however, participants can learn to correctly identify such contrasts quite quickly [7], [9], [12], [26], [27]. As shown in Figure 1, members of Group 1 experienced repeated stimulus exposure during EEG collection. Group 2 also experienced stimulus exposure; but in addition, participated in a perceptual task. The task was to identify one of the two sounds as “ba” and the other as “mba”. The experiment was designed to adhere to time lines commonly used in training literature so that P2 growth functions could be compared at similar points in time to previously published studies. This means, each group of participants was tested at four points in time; two measurements conducted on two consecutive days and then again a week later. The fourth, follow-up test was conducted months later to identify any retention pattern of perceptual and physiological changes.

We analyzed evoked activity recorded from electrode Cz to compare obtained results with the established test-retest literature. We also conducted multi-sensor analyses to characterize the distribution of evoked activity across the scalp, beyond electrode Cz, as well as to identify regions of interest (ROI) where changes in neural activity might be greatest. An additional aim was to determine if any identifiable perceptual and physiological changes would be retained. Perceptual improvements associated with stimulus identification training have been shown to be retained for periods in excess of three months [28], [29].

Results from the present study show that repeated stimulus exposure and focused listening tasks alter the physiological processing of sound. P2 changes were maximal over temporal-occipital regions of the scalp but less so over the midline central electrodes such as Cz. The effects were rapid for both groups, with enhanced synchronous activity persisting months after the last auditory experience for Group 2.

## Results

### Electrophysiology Data: Sessions 1–3

#### Peak analysis at electrode site Cz

**Figure 2:**
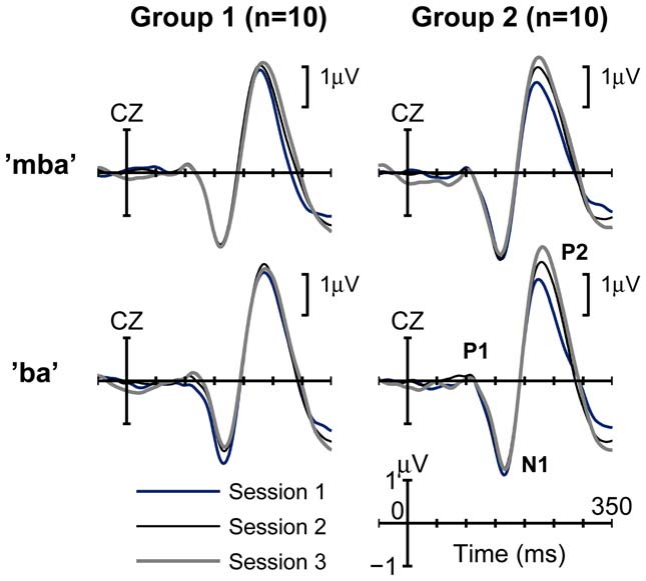
Group averaged P1-N1-P2 complexes recorded from electrode site Cz. Regardless of stimulus type (“mba” or “ba”), P2 peak amplitudes significantly increased across three sessions for Group 2 (exposure + task) but not Group 1 (exposure only). Sessions 1 and 2 were conducted on two consecutive days. Session 3 was conducted one week later.

When data were examined from electrode site Cz, the location often used in test-retest studies, there were increases in P2 amplitude for Group 2 but not Group 1 (Figure 2). A repeated measures of analysis of variance (ANOVA) for P2 *amplitude* showed a main effect of Session, (F_(2,36)_  = 10.69, *p*<0.0005, partial η^2^ = 0.37) as well as a Group x Session interaction (F_(2, 36)_ = 5.02, *p*<0.05, partial η^2^ = 0.22). Post-hoc tests for the Group x Session interaction effect revealed a simple main effect of Session in Group 2 (F_(2, 17)_ = 13.28, *p*<0.0005, partial η^2^ = 0.61), but not in Group 1 (F_(2,17)_ = 1.17, *p* = 0.33, partial η^2^ = 0.12). In Group 2, P2 peak amplitudes increased significantly from Session 1 to Session 2 (*p*<0.05), and Session 1 to Session 3 (*p*<0.0005). For P2 peak *latencies*, there was a main effect of Session (F_(2,36)_ = 3.87, *p*<0.05, partial η^2^ = 0.18) but no Group x Session interaction effect. Post hoc analyses for the main effect of Session indicated a delay in peak latency on Session 3 compared to that on Session 1 (*p*<0.05). There were no significant effects for P1 and N1 latency and amplitude.

#### Analysis with multiple electrodes using Partial Least Square (PLS) analysis

**Figure 3:**
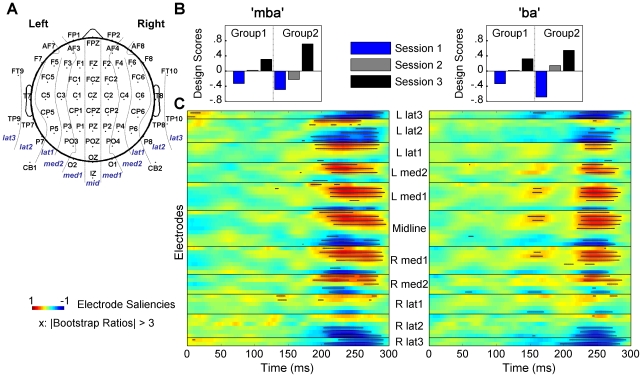
Results from Mean-Centering PLS analyses. (A) Electrode Montage. To report PLS results, Electrode locations were classified into 11 sagittal layers indicated by the dotted lines: three lateral (lat1–3), two medial layers (med1–2) in the two hemispheres, and one midline layer (mid). (B) Contrast weights identified for the first significant LV. For each stimulus type, the largest difference was observed between Sessions 1 and 3, in the P2 latency range, for both groups, but the degree of difference was greater in Group 2. (C) Spatiotemporal patterns of electrode saliencies and bootstrap results corresponding to the design LV shown in (B). The x-axis represents time in milliseconds (ms) starting at the stimulus onset marked as 0 ms. The y-axis represents electrodes organized in 11 blocks corresponding to the 11 sagittal layers in the montage shown in (A). Within each block, electrodes are ordered from top to bottom representing anterior to posterior sites. Each horizontal color bar represents temporal patterns of the electrode saliencies for a given electrode. Warm (more red) color illustrates time points with positive differences expressed in the design contrasts; cool (more blue) color expresses those of negative. Positive saliencies (warm color), and negative saliencies (cool color) indicate time points at which the amplitude of the AEP was enhanced over three experimental sessions. Saliencies are scaled with the singular value. For each electrode, horizontal black bars (comprised of individual “x”s) are plotted over the color contrasts to identify the time points at which differences expressed in the contrasts were stable across participants (bootstrap ratios >3).

**Figure 4:**
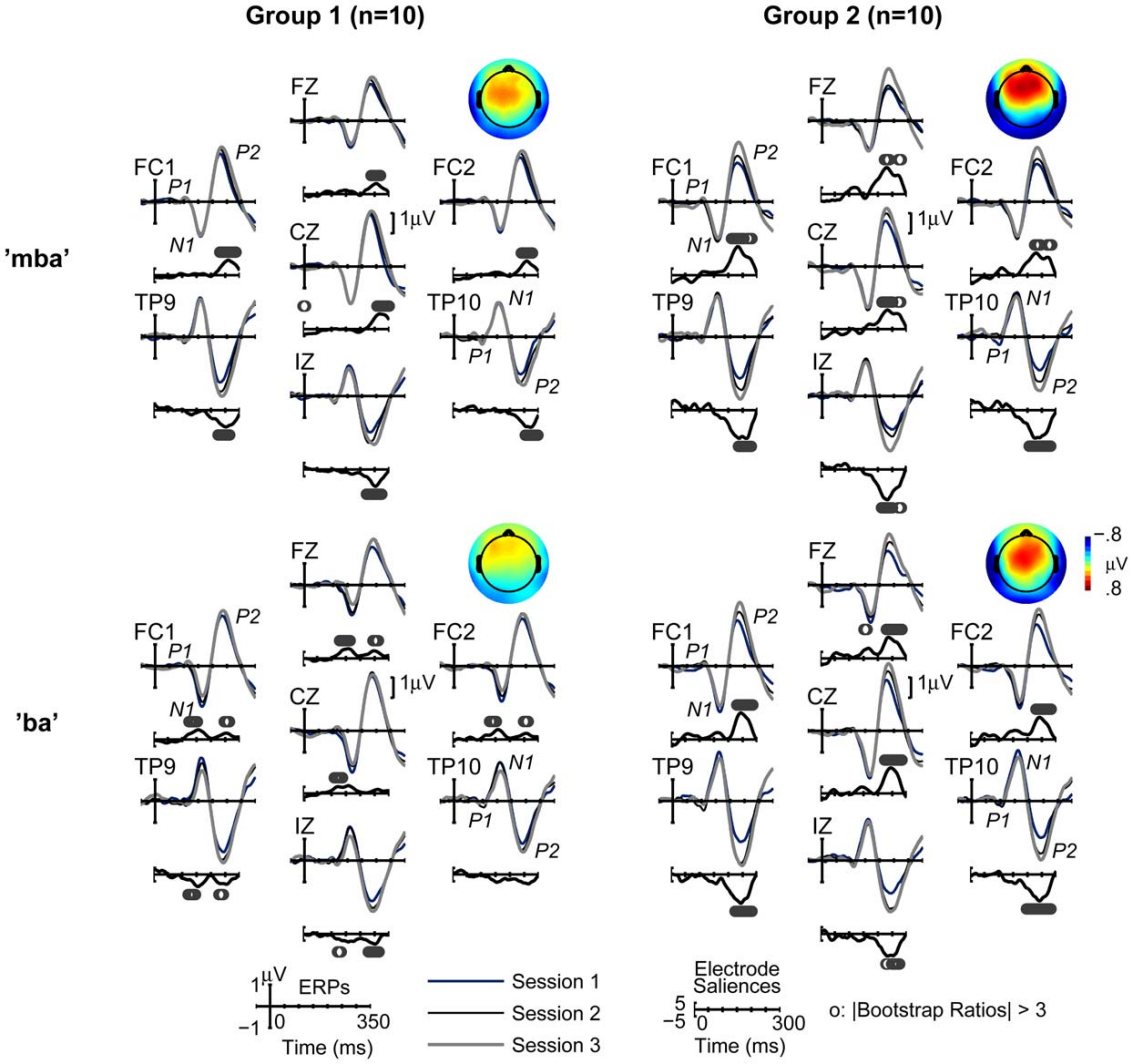
Results from Non-rotated PLS analyses and AEP waveforms for selected electrodes. Saliencies are displayed as waveforms and the circles on the top or bottom of each waveform indicate time points at which bootstrap ratios were above threshold. Larger and reliable saliencies were observed in the time range of P2 responses. Topographical maps illustrate the scalp distribution of the session effect on P2 responses. Displayed are AEP differences between Sessions 1 and 3 averaged over 190–290 ms.

A partial least square (PLS) statistical procedure [30]–[32] was performed to characterize the distribution of the AEP waveform differences (effects) observed across experimental conditions. This approach overcomes some of the disadvantages of more conventional analyses (e.g., ANOVA) performed on a single or subset of electrodes (e.g., peak analysis at Cz), which might overlook possible effects elsewhere, or might be biased by the experimenter's subjective visual inspection when deciding which electrodes should be included in the analysis [31]. PLS makes no *a priori* assumptions regarding the electrode locations and latencies of the effect, and can objectively identify *where* and *when*, within the AEP waveforms, the most reliable differences were observed across experimental conditions. Using AEP data from all sample points and from all recorded electrodes, two sets of PLS were conducted (Figures 3 and 4). First, a Mean-Centering (MC) PLS was performed for each stimulus type to assess spatio-temporal patterns of the AEP changes associated with group manipulations over three experimental sessions. These tests were exploratory with no hypothesis regarding the patterns of the effect. Second, we ran a Non-Rotated (NR) PLS, separately for each group and for each stimulus type, to specifically test a hypothesis that AEP amplitude increases linearly over three test sessions. Results showed: 1) the session effect, manifested as an increase in the AEP amplitude across three testing sessions, was present in both Group 1 and Group 2 over bilateral temporal-occipital and anterior-central areas, predominantly during the P2 response latency, and 2) this effect was larger in Group 2. The detailed reports are as follows.

Figure 3 shows the results of MC-PLS analysis. For the “mba” stimulus, MC-PLS generated six latent variables (LVs), only the first of which was significant by permutation test (*p*<*0.001*). The singular value for this significant LV accounted for 64.5% of cross-block covariance. The design scores for this LV showed contrast weights, varying in values over three test sessions across two groups, but differing most strongly between Session 1 and Session 3 in both Group 1 and Group 2, with larger difference in Group 2 (Figure 3B, left). The electrode saliencies for this LV are shown in horizontal color bars in Figure 3C (left), identifying spatiotemporal patterns of AEP difference associated with the contrasts expressed in the design scores. We observed the experimental effects at time points approximately between 190 and 290 ms, corresponding to the latency of P2 responses, broadly distributed over temporal-posterior (cool blue colors in the salience image) and anterior-central areas (warm red colors in the salience image). The onset of the differences appears to be slightly earlier at temporal-posterior sites (e.g. CB1) than those at central sites (e.g., FC1). Table 1 provides a descriptive summary of the largest reliable saliencies, identifying the electrode locations at which the strongest effects were observed. The strongest effects, indicated by largest saliencies, were observed at the inferior part of the posterior temporal electrodes in the both hemispheres (TP9, CB1, TP10, and CB2), the posterior inferior midline electrode (IZ), and the anterior part of medial to midline central electrodes (FC1, F1, F2, FCZ, and FZ). Note that largest saliencies were observed at posterior temporal and occipital areas rather than the anterior central areas.

**Table 1:** Electrodes showing the largest and reliable saliencies from MC-PLS in the P2 latency time range.

Electrode	Salience	Bootstrap Ratio
	Average score^a^	Average score^b^
***“mba”***		
TP9	1.866	4.043
CB2	1.841	4.462
CB1	1.783	3.782
IZ	1.776	4.174
TP10	1.699	4.919
F1	1.589	3.794
F2	1.580	3.879
FC1	1.545	4.056
FCZ	1.521	3.802
FZ	1.518	3.437
***“ba”***		
TP9	2.041	5.087
TP10	1.909	5.149
CB1	1.835	3.943
FT9	1.735	4.137
IZ	1.664	3.773
FT10	1.479	4.174
CB2	1.472	3.911
FCZ	1.417	4.443
F9	1.404	3.590
FC1	1.391	4.047

Note. The average scores are measured for the time points between 190 ms and 290 ms. Listed are the electrodes with the ten largest average saliencies for each stimulus condition.

a Absolute value of the average salience * 10^−2^

b Absolute value of the average bootstrap ratio

Similar results were obtained for the “ba” stimulus. Six LVs were generated, only the first of which was significant (*p*<0.001). The singular value for this significant LV accounted for 59.04% of cross-block covariance. Similar to those in “mba” condition, the design scores for this LV (Figure 3B, right) differed most between Sessions 1 and 3 in both Group 1 and Group 2, with larger differences for Group 2. The electrode saliencies for the LV (Figure 3C, right) identified experimental effects at temporal-posterior areas approximately between 190 and 290 ms, and at anterior-central areas approximately between 230 and 280 ms. The strongest effects were found at the inferior part of the temporal and posterior electrodes in the both hemispheres (FT9, TP9, CB1, FT10, TP10, and CB2), the posterior inferior midline electrode (IZ), and the anterior part of medial to midline central electrodes (FC1, FCZ, and FZ) (see Table 1). Similar to “mba” condition, these results indicated increases in P2 amplitude over three experimental sessions in both groups, with the largest increase in Group 2. Additionally in this condition, some differences were observed at anterior-central (e.g., FC1, FC2, and FZ) and anterior temporal (e.g., FT9) areas at around 130 to 180 ms, suggesting a small amount of reduction in N1 amplitudes at these locations. However, this effect was weaker, indicated by less warm or cool colors in the salience image, and was restricted to a few sets of electrodes.

The LV generated from each of the NR PLS was significant for “mba” (*p*<0.001) and “ba” (*p*<0.01) in Group 1, and for “mba” (*p*<0.001), and “ba” (*p*<0.001) in Group 2. Figure 4 shows electrodes saliencies and bootstrap results of these contrast tests along with AEP waveforms that theses analyses were based on. Results are shown for Cz as well as a small set of electrodes, representing the major scalp areas (the temporal-occipital and the anterior-central areas) that showed the largest effect in the results of MC-PLS analyses presented earlier (c.f., Table 1). Reliable saliencies (above bootstrap threshold) were most prevalent in the P2 latency range for “mba” conditions in Group 1 and both “mba” and “ba” conditions in Group 2. The saliencies were also smaller for Group 1 and appeared after the P2 peak, on the down slope, especially at Cz and anterior-central areas.

#### Peak analysis at selected electrode within ROIs

**Figure 5:**
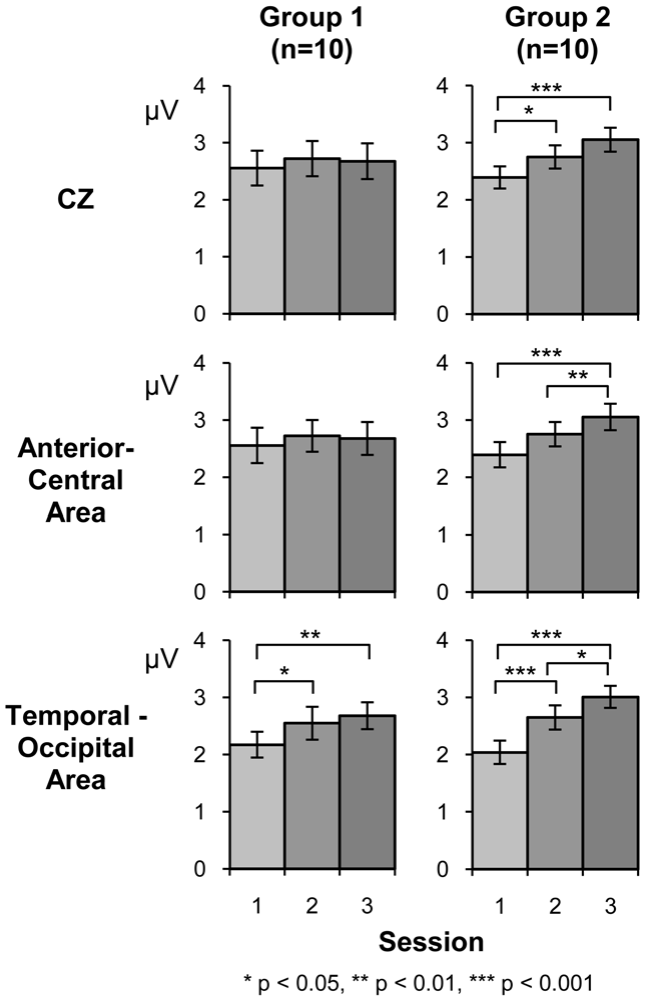
P2 amplitude changes across Sessions at three ROIs. Increases in P2 amplitude were most prevalent within the temporal-occipital region for both Groups across sessions. This session effect was not observed at anterior-central areas as well as at vertex (Cz) for Group 1.

According to PLS analyses, temporal-posterior and anterior-central areas showed the strongest experimental effects. To allow direct comparison to the single electrode analysis at Cz reported earlier, electrodes within these two regions of interest (ROI) were selected so that P2 peak analyses could be performed. The selected electrodes included FC1, FZ, and FC2 electrodes for the anterior-central area, and TP9, IZ, and TP10 electrodes for the temporal-occipital area. Results are summarized in Figures 4 and 5.

For the anterior-central area, the group averaged AEP waveforms (Figure 4) show enhanced P2 peak amplitudes over three sessions for Group 2, that are less apparent for Group 1 

[main effect of Session (F_(2, 36)_ = 16.92, *p*<0.0005, partial η^2^ = 0.48), Group x Session interaction effect (F_(2,36)_ = 3.47, *p*<0.05, partial η^2^ = 0.16)]. Post-hoc tests for the Group x Session interaction effect revealed a significant simple main effect of Session in Group 2 (F_(2, 17)_ = 11.72, *p*<0.001, partial η^2^ = 0.58), while the effect was only marginal in Group 1 (F_(2,17)_ = 2.80, *p* = 0.065, partial η^2^ = 0.28). P2 peak amplitudes in Group 2 increased from Session 1 to Session 3 (*p*<0.0005), and from Session 2 to Session 3 (*p*<0.005). For P2 peak latencies, there was a main effect of Session (F_(2, 36)_ = 4.28, *p*<0.05, partial η^2^ = 0.19) with latency being delayed on Session 3 compared to that on Session 1(*p*<0.05).

For the temporal-occipital area, group averaged AEP waveforms (Figure 4) showed enhanced P2 peak amplitudes across each session for both Groups. For P2 peak amplitudes, ANOVA revealed a main effect of Session (F_(2,36)_ = 32.68, *p*<0.0001, partial η^2^ = 0.65), showing increases in P2 amplitude on Session 2 (*p*<0.0005), and on Session 3 (*p*<0.0001), compared to Session 1. P2 amplitude for Session 3 was also significantly larger than Session 2 (*p*<0.05). A Group x Session interaction effect was only marginal (F_(2,36)_ = 3.09, *p* = 0.059, partial η^2^ = 0.15) because the session effects were observed in both Group 1, (F_(2,17)_ = 6.54, *p*<0.01, partial η^2^ = 0.44), and Group 2 (F_(2,17)_ = 22.55, *p*<0.0001, partial η^2^ = 0.73). For Group 2, P2 amplitudes increased on Session 2 (*p*<0.001), and on Session 3 (*p*<0.0001), compared to those on Session 1, and from Session 2 to Session 3 (*p*<0.05). In Group 1, P2 amplitudes increased on Session 2 (*p*<0.05), and on Session 3 (*p*<0.01), compared to those on Session 1. For P2 peak latencies, there were no significant effects at the temporal-occipital area.

Figure 5 summarizes changes in P2 peak amplitude over three test sessions at three scalp locations; vertex (Cz), the anterior-central area, and the temporal-occipital area. The greatest amount of P2 amplitude growth took place over the temporal-occipital area in both groups, with the largest amount of growth between Session 1 and Session 3. P2 amplitude growth was also observed at the anterior-central area and at Cz in Group 2, but was absent in Group 1. In general, P2 peak latency was delayed between Session 1 and Session 3 when measured at anterior-central and Cz, but not at the temporal-occipital area.

### Electrophysiology Data: Retention

**Figure 6:**
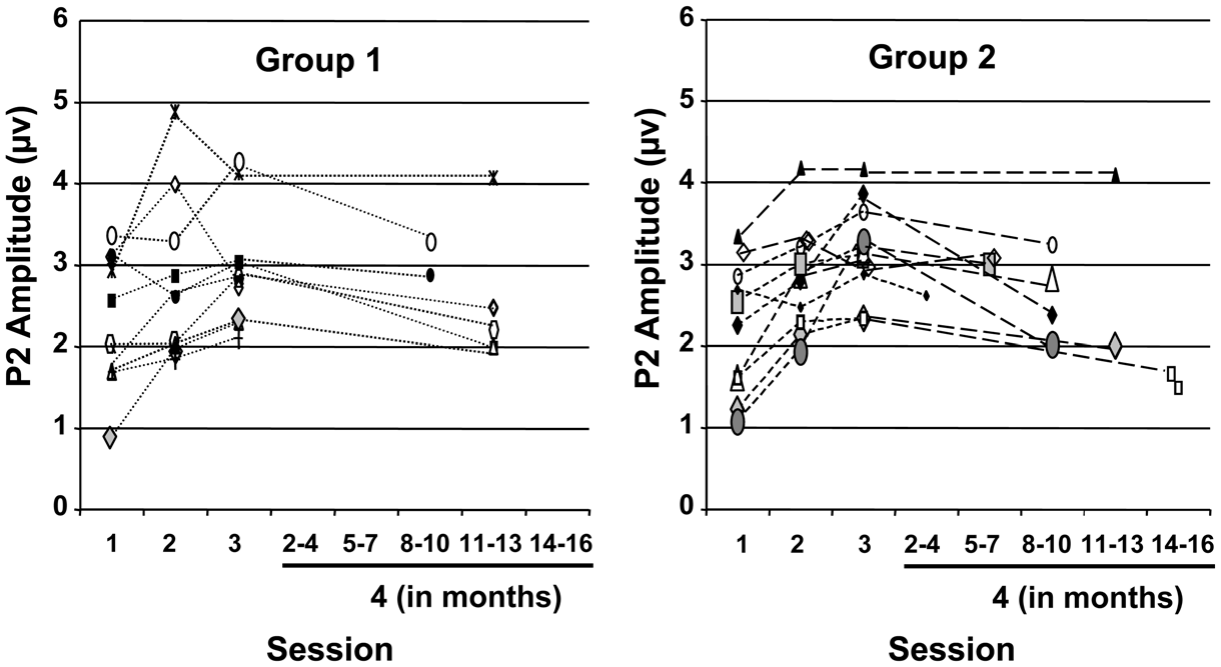
Retention Data. Individual P2 peak amplitude data are shown for all four Sessions in response to the stimulus “mba”. Results shown are from electrode site TP9. When looking at individual subjects, enhanced P2 amplitudes can be seen for many individuals (in Group 2) even though they had not heard these sounds for many months.

Converging evidence from PLS and ROI analyses showed the largest changes in P2 amplitude in the temporal-occipital area. Therefore, physiological retention patterns from this region were analyzed. When comparing the Session 4 to Session 3, there was a decrease in P2 amplitude, reflected in the main effect of Session (F_(1,15)_ = 8.97, *p*<0.01, partial η^2^ = 0.37). However, P2 amplitudes at Session 4 did not return to their starting point (Session 1) (F_(1,15)_ = 11.72, *p*<0.005, partial η^2^ = 0.44). When examining the Session x Group interaction (F_(1,15)_ = 6.68, *p*<0.05, partial η^2^ = 0.31), P2 amplitude growth was retained for participants in Group 2 (*p*<0.0005), but not in Group 1 (*p* = 0.592). Representative data from the temporal-occipital region, where the effect was largest (electrode site TP9), are shown in Figure 6. Although P2 latency increased from Session 1 to Session 3, when measured at CZ and anterior-central area, these latency changes were not retained.

### Behavioral Results

**Figure 7:**
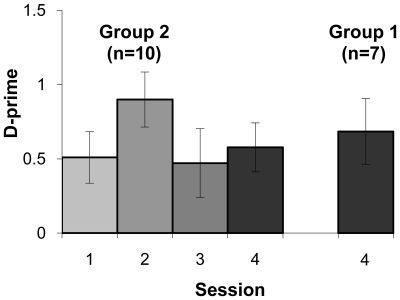
Group d-prime scores and standard error bars are shown for each test session.

Only Group 2 participated in intervening tasks between each EEG recording session and there was no significant change in perception across Sessions 1 through 3 (F_(2,18)_ = 1.99, *p* = 0.166, partial η^2^ = 0.18). However, as shown in Figure 7, there was an increase in performance within the first 24 hours, from Session 1 to Session 2, according to a one-tailed paired t-test (t _(9)_ = 1.80 *p* = 0.052). The retention scores were not significant when comparing Session 1 to 4 (t _(9)_ = 0.29, *p* = 0.782) and Session 3 to 4 (t _(9)_ = 0.55, *p* = 0.599). Members of Group 1 who were tested behaviorally following their final EEG recording session showed no significant differences in the ability to correctly identify “mba” and “ba” at Session 4 when compared to Group 2 (t_(12)_ = 0.39, *p* = 0.706).

To evaluate the relationship between the perceptual data and the P2 amplitudes, we computed correlations between each participant's d-prime and P2 amplitude for within and across sessions. P2 amplitudes were quantified as the average ERP amplitudes between 190 ms and 290 ms at two locations; CZ as a electrode site commonly used in the literature, and temporal-occipital ROI, where the session effect was robust. None were significant.

## Discussion

There is an abundance of literature demonstrating that auditory training can alter the physiological encoding of auditory stimuli but little is known about the contribution of repeated stimulus exposure and tasks to the reported training effects. These issues are important when defining normal processes associated with auditory learning and when developing effective training programs aimed at rehabilitating impaired perception.

Our findings show that mere repeated stimulus exposure alters the way sound is encoded in the brain, and these alterations are reflected by enhanced evoked brain activity in the same P2 latency range previously identified as being responsive to auditory training. However, the effects of exposure and engaged activity may be different from one another. When stimuli were paired with a task the effect on the evoked response was even greater. With the additional task, growth effects were rapid and long lasting, with enhanced synchronous activity persisting months after the last auditory experience. The effect was cumulative, with the magnitude of the auditory evoked P2 peak increasing with each additional listening experience. P2 growth was maximal over temporal-occipital regions of the scalp and less so over central (e.g., Cz) areas. Despite observing enhanced neural response patterns, especially when sound was paired with a listening task, enhanced P2 amplitudes did not coincide with measurable improvement in perception. We therefore speculate that the cumulative effect likely involves auditory memory involving sound recognition; but in the absence training, the observed physiological changes are insufficient to result in changes in learned behavior.

### Scalp Distribution of P2 Amplitude Growth

In our initial analysis we examined P2 amplitudes from the vertex electrode site (Cz). The purpose of examining this electrode in isolation was to compare our results to the published test-retest literature, because changes in P2 amplitude across test sessions had not previously been reported. The P1-N1-P2 complex is typically largest over vertex, so it is understandable why this electrode location is so often researched and used in clinical situations when it is neither feasible nor practical to apply a large number of electrodes. However, it is also important to consider phenomena that might be occurring outside this region as well as defining scalp locations where experience-related changes in the central auditory system are most visible.

When evoked brain activity from electrode site Cz was examined, P2 amplitude did not change from session to session for Group 1 (the group merely exposed to the stimuli). These results are similar to those reported in the test-retest literature, and help to explain why significant changes in peak amplitudes were not reported when only a subset of midline electrodes had been examined. However, prior stimulus exposure does appear to have an effect on brain responses if areas other than vertex are examined. Group 1, for example, showed significant increases in P2 amplitude across test sessions from electrodes located over temporal-occipital regions.

Rather than selecting specific electrode sites for examination, which can be subjective and possibly overlook significant findings at other electrode locations, we explored multi-sample AEP segments from all electrodes on the scalp and identified spatiotemporal patterns of AEP differences observed across experimental conditions. To accomplish this, a multivariate statistical approach was used to identify time points and regions of interest on the scalp for further analysis. Using these methods, the largest differences in AEPs from session-to-session were found over temporal-occipital regions of the scalp and least so over central locations (e.g., Fz and Cz). This distribution of AEP change, across sessions, was similar for both groups, meaning the effects of stimulus exposure and exposure-plus-task were similar in that they were most evident over temporal-occipital regions of the scalp. But the magnitude of change was different between groups. We speculate that the P2 growth seen in Group 2 at vertex reflects enhanced neural activity from adjacent areas in temporal lobes. In turn, the absence of observable P2 growth at vertex for Group 1 could reflect growth over temporal-occipital regions that was insufficient to be detected at vertex. An alternative interpretation is that different brain sources [33], and/or sources with different orientations in space and time [34], are activated in an active listening task compared to passive listening.

### Timeline and Retention of Enhanced P2 Amplitudes

Enhanced P2 amplitudes occurred rapidly and were long-lasting, with enhanced synchronous activity persisting months after the last auditory experience. With the number of days between test sessions being strictly controlled for, it can be said that P2 amplitude growth is greater when there is a shorter period of time between sessions. In the temporal-occipital region, the amount of growth was greater within the first 24 hours than when separated by a week (e.g., Session 2 vs. 3). And despite not hearing these sounds for extended periods of time, enhanced P2 amplitudes were seen a week later for both groups. The most compelling effect; however, involved Group 2 where P2 amplitude enhancements could be seen approximately a year following the last listening experience.

These retention patterns motivate us to question the role of memory and if enhanced P2 amplitudes represent some form or pre-attentive correlate of sound recognition that is influenced by time. Because the magnitude and retention of P2 change was larger for Group 2 compared to Group 1, it is possible that participating in the listening task strengthens the effect by inducing some type of meaning, category, or purpose to the otherwise irrelevant sounds. If so, P2 might reflect automatic stimulus recognition based on prior stimulus experience.

### Learning and Memory

Stimulus repetition paradigms have been used to probe functional characteristics of neural populations associated with attention, learning, and memory. Passive exposure to tone pips, for example, can result in physiological changes in the primary auditory cortex of adult cats and can persist for several months [35], [36]. In the visual system, repeated stimulus experience can lead to both short- and long-term enhancement and suppression of neuronal responses in subpopulations of visual neurons [37]. Visual repetition suppression, sometimes described in terms of adaptation, habituation, or neural priming, appears to be an intrinsic property of visual cortical areas such as inferior temporal cortex and is thought to be important for perceptual learning and priming [37], [38]. In contrast, enhancement of neuronal responses, in the same 200 ms latency range explored here, have been shown to be enhanced for objects with learned behavioral relevance and is said to depend on feedback to temporal cortex from prefrontal cortex and is important for working memory [39].

Adaptation and habituation patterns of human auditory evoked P1-N1-P2 responses have also been examined using stimulus repetition paradigms and it is well documented that the N1 component shows rapid declines in amplitude, within minutes of initial stimulation [25], [40], [41]. Recovery of the N1, on subsequent days, is also well documented which explains reports of good test-retest reliability here and elsewhere. In contrast, P2 amplitudes remain almost constant within a recording session, but show enhancement on subsequent days [25]. Therefore, our interpretation is that the N1 reflects adaptive type tendencies, similar to those described in the visual stimulus repetition paradigms, and P2 enhancement reflects a consolidation process associated with auditory memory and learned relevance. In other words, in auditory circuitry with specific neuronal subpopulations, immediate N1 suppression might reflect feed-forward responses that evolve and contribute over time to top-down connections that consolidate and contribute to the observed P2 enhancement. The important point here is that changes in N1 and P2 follow different time courses. When repeated stimulus exposure is combined with a training task, the resultant interplay of neurons could result in coincident changes in P2 and perception. According to source analyses, this complex interplay between excitatory and inhibitory connections could involve regions anterior to the first transverse gyrus of Heschl in auditory cortex for P2, and regions posterior to Heschl's gyrus contributing to N1 [25].

It is also reasonable to assume that the group effects reported here are a mere byproduct of the fact that the task contained 50 additional stimulus presentations, a confound that is difficult to avoid if one wants to study the additional effects of a listening task. It seems unlikely however that this modest amount of stimuli could have contributed to the group effects reported here since our prior studies have shown that increments of 25 and 50 stimuli, presented on the same day, do not result in significant increases in P2 amplitude [25]. Nevertheless, regardless of what the specific contributing mechanisms might be, some concluding statements can be made based on converging evidence from prior training experiments. First, P2 enhancement resulting from either stimulus exposure or task execution is similar in morphology to that reported in our prior training experiments; however, the magnitude of change appears to be less than that reported in prior training experiments [9]. Second, unlike the stimulus-specific P2 effects seen with training [9], [12], the effects of mere stimulus exposure do not appear to be stimulus specific. Ross and Tremblay [25] reported comparable increases in P2 amplitude in response to a noise stimulus that participants were exposed to, but not part of a listening task. Third, regardless of whether auditory experience involves mere stimulus exposure or training, not all individuals exhibit physiological changes. Despite similar auditory experiences, the central auditory systems of individuals appear more or less responsive to listening experience and such heterogeneity might be informative when probing the differences between learners and non-learners who undergo various types of auditory training exercises [9]. For example, the auditory systems of older adults appear to be less responsive to prior stimulus experience [25].

Because physiological changes did not coincide with perceptual improvements we interpret these findings to suggest that the AEP enhancement patterns observed here may reflect sound recognition that builds up over time, and is part of the learning experience. What is more, the disassociation between physiology and perception observed in this experiment might be explained by the time course of brain-behavioral changes. Previous behavioral evidence indicates that mere exposure to sounds improves performance in subsequent recognition and identification tasks [42]–[44]. There is also evidence that brain-behavior systems do not share the same time course of change [29], [45], [46]. We therefore question if participating in the behavioral tasks, within the week that separated sessions 2 and 3, might have resulted in modest perceptual gains because the ability to correctly identify the two stimuli changed in a positive direction within the 24 hours that separated Sessions 1 and 2.

Another variable is attention. When AEPs are recorded while the subject is actively attending and executing the training task it provides an opportunity to characterize neural processes that are active during learning. As such, Alain et al. [12] identified latency regions following the P2 peak that coincided with improved perception of the same VOT contrast reported here. For this reason, examining sustained activity, and even oscillatory activity, might yield brain-behavior associations not reported here.

In conclusion, we propose that enhanced P2 activity reflects sound recognition that builds over time, and is part of the learning experience. We speculate that repeated stimulus exposure primes the auditory system in a general way that is not stimulus specific and can be recalled following a long period of time. In contrast to exposure, training exercises shape the system such that the acoustic distinctions that make specific sounds relevant are reinforced, and perceptual gains can be made.

## Methods

### Ethics Statement

Ethics approval for this experiment was obtained by the University of Washington Institutional Review Board and participants provided their written informed consent using a University of Washington approved consent form. All research was conducted according to the principles expressed in the Declaration of Helsinki.

### Participants

Twenty right-handed native speakers of English participated in this experiment. The ten participants, who were randomly assigned to Group 1 (male  = 5, female  = 5), ranged in age from 22 to 39 years (mean  = 29). Group 2 was comprised of people who ranged in age from 18–39 years (mean  = 25; male  = 2, female  = 8). All participants had normal audiometric thresholds; better than 25 dB HL in the frequency range of 250 through 8000 Hz bilaterally. They were in good general health with no history of otological or neurological disorders.

### Stimuli

Auditory stimuli were two versions of the Klatt synthesized syllable “ba” [47]. The two stimuli were identical in their duration (180 ms) and spectral content but differed in voice onset time (VOT). One stimulus had a VOT of −20 ms (denoted as “mba”) while the other had −10 ms (denoted as “ba”). Adult native speakers of English routinely identify these two pre-voiced stimuli as “ba” [26]; however, following training, they can learn to differentiate the two sounds and correctly identify −20 ms and −10 ms VOT sounds as “mba” and “ba”, respectively [7]–[9], [25], [27], [46]. Because these two “ba” stimuli are the same tokens used in our previous experiments, additional descriptions of the stimuli can be found in our previous publication [27]. A brief period of silence precedes each sound (approximately 50 ms for the −20 ms stimulus; 60 ms for the −10 ms VOT token) and thus AEP latencies are delayed by this same amount of time.

### Procedure

Group 1 participated in the EEG sessions only and did not partake in the intervening perceptual task; that is, they were passively exposed to homogenous trains of auditory stimuli during each of the four EEG sessions. The procedure was the same for Group 2, except intervening behavioral tasks took place at the end of each auditory evoked potential (AEP) session. Each group of listeners was tested on four separate occasions (Figure 1). The timeline of testing was strictly controlled with each participant being tested on two consecutive days (Session 1 and Session 2), and again one week later (Session 3). All participants had at least a two month break before being asked to participate in the retention session (Session 4). A limited number of participants could return for testing at different points in time with the number of days, since Session 1 testing, averaging: 378 days for Group 1 (range = 308 to 419 days, n = 7); and 287 days (range  = 87 days to 461 days, n  = 10) for Group 2. The test dates for Session 4 were intentionally spread across a broad time period so that different retention times could be evaluated.

#### Perceptual testing

Group 2 participated in a two-alternative forced-choice identification task. Instructions were provided in print to each subject. They were: “You will hear some sounds and I want you to label the sounds as you perceive them. You will label the sounds based on two choices that will be displayed on the computer monitor. I want you to label the sounds you hear using only the left button on the mouse. There is no right or wrong answer; it's simply your perception of what you hear.”

Participants sat in front of a computer monitor displaying two labels, “mba” and “ba”. They were then presented with 50 trials of randomized stimulus sounds (25 of “mba”, 25 of “ba”) binaurally at a level of 76 dB SPL using Etymotic Research (ER3a) insert earphones. The test was administrated in a self-paced fashion in which participants indicated their judgments after each sound presentation, and the mouse click response triggered the presentation of the next sound. Feedback was not provided to the participants.

#### Electrophysiological testing

Auditory evoked potentials (AEPs) were recorded while participants were watching a closed-captioned silent (muted) movie while passively hearing the stimuli. Stimuli were presented in two blocks of 400 trials, separated by a five-minute break. The same sound was presented in repetition (ISI  = 1993 ms) within a block. For Sessions 1 and 2, the order of the stimulus presentation was counter-balanced across groups such that, for a given test session, half of the participants in each group heard “mba” in the first block followed by “ba” in the second block, and the other half heard them in the reverse order. Stimulus order was not strictly counterbalanced for Sessions 3 and 4. Stimuli were presented to the right ear at 76 dB SPL using the same ER3a insert earphones used during perceptual testing.

### Electrophysiology Recording and Analysis

Continuous EEG signals were recorded from 59 electrodes embedded in an elastic cap (Electro-cap International, Inc.) using a PC-based Neuroscan System (SCAN, ver. 4.3.3) with SynAmps2 amplifiers. Electrode montage followed the extended 10–20 system and is shown in Figure 3A. Four additional electrodes were placed on the inferior and outer canthus of each eye to monitor eye blink activity. EEG signals from above electrodes were referenced to a common electrode on Vertex (Cz), analog bandpass-filtered between 0.15 Hz and 100 Hz (12 dB/octave roll off), amplified with a gain set at ×500, and converted from Analog to Digital at a sampling rate of 1000 Hz.

Offline, continuous EEG signals were epoched from 100 ms prestimulus to 500 ms poststimulus for each trial, baseline-corrected with prestimulus measures, and averaged for each stimulus condition. Epochs contained artifacts exceeding +/−70 microvolts were removed prior to averaging. The obtained signals (AEPs) were filtered with an analog simulation bandpass filter 1.0 Hz (24 dB/octave) to 20 Hz (12 dB/octave). AEP signals were then re-referenced to the average signals recorded from all electrodes, excluding those on the eyes.

AEPs for the first three sessions were analyzed to determine how repeated stimulus exposure and intervening focused listening tasks altered participants' electrophysiological responses to the stimulus sounds. To examine the retention of physiological changes, data from Session 4 were analyzed separately.

#### P2 Peak responses analysis at Cz and at selected electrodes

AEPs measured at Cz and at selected electrodes over the two scalp regions (FC1, FZ, and FC2 in the anterior-central area; TP9, IZ, and TP10 in the temporal-occipital area) were examined for changes in the peak amplitudes and latencies of the P2 response. Peak amplitude was defined as the maximum amplitude of each component relative to the pre-stimulus baseline, and peak latency as the latency of the peak amplitude from the stimulus onset. Neuroscan software was used to detect the maximum or the minimum amplitudes and the latencies of these amplitudes corresponding to the time ranges of each observed AEP peak. Peak locations were then confirmed manually. Statistical analyses on these measures were performed separately for amplitude and latency measures using a repeated-measures analysis of variance (ANOVA) implemented in SPSS software (SPSS, Inc., Chicago, USA). For example, P2 peak amplitude at Cz was analyzed with a three-way repeated measures ANOVA, with Group (Group 1 or Group 2) as a between-subject factor, and Stimulus type (“mba” or “ba”) and Session (Session 1, Session 2 or Session 3) as within-subject factors. P2 peak responses at selected electrodes were analyzed separately for the two scalp regions, with a four-way repeated measures ANOVA, with one within-subject factor, Electrode, added to the design used for Cz analysis. The factor, Electrode, had three levels, representing three electrodes included for each area. The significant results (*p*<0.05) concerning the main effect of Session and its interaction effects with other factors (Group, Stimulus type, and Electrode) are reported. Greenhouse-Geisser correction for inhomogeneity of variance was applied for all repeated measures where the degree of freedom in the numerator was greater than one [48]. Reported are uncorrected degrees of freedom with Greenhouse-Geisser adjusted *p*-values. Partial eta squared (η^2^) are also reported as estimates of effect size for the Session and its interaction effects. For post-hoc analyses, we performed simple effect tests for interaction effects and pairwise comparisons for main and simple main effects of the factors with more than two levels. Bonferroni adjustments were applied for multiple comparisons.

#### Partial least square (PLS) analysis

PLS, a multivariate statistical procedure, [30]–[32] was employed to asses the scalp distributions and the timing of AEP waveform differences associated with three experimental sessions across two groups for the two stimulus sounds. PLS finds latent variables (LVs) that explain variability observed in the dependent measures (AEPs), which directly covary with experimental conditions (e.g., groups, sessions). Using data from all electrodes, PLS makes no *a priori* assumptions regarding expected time points, or electrode locations, and identifies AEP waveform differences across conditions resulting from changes in amplitude, and/or latency of particular AEP components, or responses. The results from PLS analyses were then used to identify major scalp regions where the strongest experimental effects were observed. PLS analyses were conducted using Matlab code, developed by McIntosh, Chau, Lobaugh, & Chan, available at http://www.rotman-baycrest.on.ca. Full descriptions of this method are found in McIntosh [32] and Lobaugh et al. [31].

Two sets of PLS were used: Mean-centering PLS (MC-PLS) and non-rotated PLS. Mean-Centering PLS (MC-PLS) analyses were first performed with no *a priori* hypothesis regarding the potential pattern of effects. PLS analysis is sensitive to latency and amplitude changes. Because the acoustic content of the two stimuli differ by 10 ms of pre-voicing, the resultant P1-N1-P2 responses also reflect this stimulus related latency difference. To avoid detecting two stimuli's obligatory latency differences related to the acoustic content of the signal, instead of the experimental effects, PLS analysis was performed separately for each stimulus condition.

An AEP-amplitude data matrix was first created for each group for a given stimulus sound, with the row corresponding to participants and the columns corresponding to AEP amplitude as each sampling point from 0 ms to 300 ms within 59 electrode sites. The time range was selected to cover all post-stimulus time points up to the end of the deflection of the P2 component. Means were computed for each column for each group and subtracted from the grand mean, resulting in a mean-centered derivation matrix. Singular value decomposition (SVD) was performed on the mean-centered matrix with an orthogonal design matrix contrasting two groups over three experimental sessions. This procedure produced six orthogonal latent variables that contributed to the AEP waveform differences. Three outputs were generated for each latent variable within each mean-centering PLS analysis; 1) a singular value that expresses the proportion of covariance that the LV was accounted for, 2) design salience that represents weights of the contrast for the LV, and 3) electrode saliencies that show spatiotemporal patterns of the contribution made by the LV. The strength of LVs (statistical significance) was assessed using permutation tests (500 permutations), and the stability of the differences observed (reliability of saliencies identified on the LV) were determined by bootstrap tests using 500 samples.

NR-PLS analysis was performed separately for each stimulus within each Group, to determine if there were increases in AEP amplitude over three sessions. The contrast weights were (−1 0 1) with condition ordering Session 1, 2 and 3. SVD was performed for each test (4 tests for each of the group x stimulus type conditions), yielding one non-orthogonal latent variable per test. Electrode saliencies for the given contrast weights were generated. Statistical assessments of the LVs and reliability of electrode saliencies were determined in the same way as mean-centering PLS analysis described above.

#### Retention Analysis

To evaluate retention the observed P2 amplitude growth, we examined relative changes in P2 peak amplitudes measured on Session 4 and contrasted it with those measured on Session 3 and on Session 1 (baseline) measured at temporal-occipital area. Four repeated measures of ANOVA were performed with Group as a between-subject factor, and Session, Stimulus Type and Electrode as within-subject factors. The two levels of Session factor were either Session 1 and Session 4, or Session 3 and Session 4. The factor, Electrode, had three levels for three selected electrodes described earlier (TP9, IZ, and TP10).

### Behavioral Data Analysis

To assess perceptual performance in Group 2, *d*-prime scores were computed for each participant. Correct identification and correct rejection were scored if participants labeled “mba” for −20 ms VOT sound and “ba” for −10 ms VOT sound, respectively. A one-way repeated measures ANOVA with Session as a within-subject factor (Session 1, 2, and 3) was performed.
